# Factors associated with the level of physical activity in a multi-ethnic pregnant population – a cross-sectional study at the time of diagnosis with gestational diabetes

**DOI:** 10.1186/s12884-021-04335-x

**Published:** 2022-01-03

**Authors:** L Garnweidner-Holme, L Henriksen, K Bjerkan, J Lium, M Lukasse

**Affiliations:** 1 Faculty of Health Sciences, Department of Nursing and Health Promotion, Oslo Metropolitan University, Oslo, Norway; 2 Division of General Gynaecology and Obstetrics, Oslo University Hospital, Oslo, Norway; 3 Department for diabetes, Aker Hospital, Oslo University Hospital, Oslo, Norway; 4 Faculty for medicine, University of Bergen, Bergen, Norway; 5 Faculty of Health and Social Sciences, University of South-Eastern Norway, Campus Vestfold, Notodden, Norway

**Keywords:** Leisure-time physical activity, Pregnant women, Gestational diabetes mellitus, Pregnancy Physical Activity Questionnaire

## Abstract

**Background:**

Regular physical activity during pregnancy can prevent several adverse health outcomes during this period of a woman’s life. Previous studies have shown that many women do not meet national recommendations for physical activity. This study aims to examine factors associated with sufficient leisure-time physical activity (LTPA) in a multicultural sample of pregnant women recently diagnosed with gestational diabetes mellitus (GDM).

**Methods:**

We performed a cross-sectional study among 238 pregnant women. The women were recruited at diabetes outpatient clinics in the Oslo region of Norway from October 2015 to April 2017. The participants reported their activity levels using the Pregnancy Physical Activity Questionnaire (PPAQ). Pearson’s chi-square tests were used to assess differences in socio-demographic, health and pregnancy-related characteristics in relation to sufficient and insufficient LTPA, and logistic regression modelling was employed to predict the likelihood of insufficient LTPA.

**Results:**

Less than half of the women in the sample (44.5%) had sufficient LTPA according to the minimum of ≥600 Met minutes per week. The majority of women were motivated to be physically active during pregnancy (84.9%). A low joint family income and being over 38 years of age increased the odds of not having sufficient LTPA. Women with sufficient LTPA had significantly higher scores of perceived health (*p* = 0.007).

**Conclusions:**

The study indicates that pregnant women need to be better informed about the positive effects of physical activity on individually perceived health. To address the low levels of LTPA among pregnant women, communication strategies must be tailored towards women with low socio-economic backgrounds.

**Trial registration:** https://clinicaltrials.gov/ct2/show/NCT02588729

**Supplementary Information:**

The online version contains supplementary material available at 10.1186/s12884-021-04335-x.

## Background

Regular physical activity during pregnancy can have a preventive effect on the development of gestational diabetes mellitus, excessive maternal weight gain [1] and other adverse pregnancy outcomes such as caesarean section [2] and preeclampsia [3] and complications during labour [4]. In addition, there is emerging evidence on the correlation between pregnant women’s level of physical activity and their quality of life [5].

A review of international guidelines for physical activity during pregnancy in women with uncomplicated pregnancies and women with GDM in the period 2000–2018 shows that most countries included in the review recommend moderate intensity exercise for at least 60–150 minutes per week [6]. Norwegian guidelines recommend that pregnant women should engage in moderate-intense physical activity for at least 150 minutes per week [7]. Physical activity can be calculated as metabolic equivalent tasks (MET), and ≥600 MET minutes a week is the recommended minimum of physical activity for adults [8]. Despite the benefits of physical activity during pregnancy, several studies show that pregnant women do not adhere to the national recommendations for physical activity [9–12]. For instance, in a large cohort study, only 14.6% of women in mid-pregnancy in Norway engaged in exercise ≥ 3 times a week, >20 min at moderate intensity [9]. In the USA, 23–29% of pregnant women were found to meet the recommendations [13]. Women generally decrease their physical activity as pregnancy progresses [14] and spend the majority of their day being sedentary (up to 60%), as shown by motion sensor data from the USA [13].

Although our society is becoming increasingly multicultural, studies about the level of physical activity among women from different ethnic backgrounds in high income countries are scarce [10, 15, 16]. The few studies that encompass a multi-ethnic population show lower levels of physical activity among ethnic minority groups. A review of the literature on levels of physical activity and exercise among pregnant women in Africa found that the participation of pregnant women in physical activity was low and declined in step with advancing pregnancy [17]. The level of physical activity was also found to be low in pregnant women in China [18] and Taiwan [19].

A large body of the literature describes pregnant women’s attitudes and barriers to and enablers of physical activity [20–22]. Barriers to physical activity are often intrapersonal, such as fatigue, lack of time and pregnancy discomforts. Frequent enablers include maternal and foetal health benefits, social support and pregnancy-specific programmes. Few environmental factors were identified [20]. Little information is available about such attitudes and barriers to and enablers of physical activity for pregnant women with GDM who are at risk from inactivity [20, 23]. A qualitative study conducted among 27 pregnant women in Australia found that women with GDM wanted clear and practical messages from credible sources about physical activity during pregnancy [23]. They asked for specific information about safe physical activity during a GDM pregnancy. The present study assesses factors associated with sufficient levels of leisure-time physical activity (LTPA) in a multi-ethnic pregnant population at the time the women were diagnosed with GDM.

## Methods

We performed a cross‐sectional study with baseline data from the Pregnant+ study, a randomised controlled trial (RCT) among pregnant women with GDM [24] (ClinicalTrials.gov/NCT02588729). Data were collected from October 2015 to April

2017 at five diabetic outpatient clinics (DOCs) in the Oslo region of Norway. Participants were recruited consecutively as they came to the DOC. To be included in the study, the women had to have a smartphone, be 18 years or older and be at a gestational age of less than 33 weeks. The women also had to be capable of filling out the questionnaire in Norwegian, Somali or Urdu. Only 14 women filled out the questionnaire in either Urdu or Somali. Participants were excluded from the study if they had a twin pregnancy. In addition, women with celiac disease or lactose intolerance were excluded since they need to follow special diets [24]. Health professionals at the DOCs identified pregnant women with GDM and checked their eligibility for participation in the study. Of 774 participants assessed for eligibility, 238 participated in the study. All of the women were diagnosed with GDM after performing a two‐hour oral glucose tolerance test (OGTT) ≥ 9 mmol/L. The OGTT consisted of a fasting blood glucose sample followed by drinking a beverage containing 75 g of anhydrous glucose and a second blood glucose sample measured 2 hours later. The definition of GDM was in accordance with the national guidelines for antenatal care and that of the WHO [25, 26].

## Measures

The participants answered a questionnaire using an electronic tablet at their first consultation at one of the DOCs included in the study. Participants were asked to report their LTPA levels during the last four weeks prior to being diagnosed with GDM using the Pregnancy Physical Activity Questionnaire (PPAQ) [27]. We have chosen four weeks to minimize a sudden effect of the diagnosis on women’s behaviours. In addition, the questionnaire contained questions about thepregnancy, including gestational age and parity, and socio-demographics including country of birth, Norwegian language skills and economic status, motivation for being active and reasons for not being active. The women were also asked to assess their general health and dietary habits [24].

## Variables

The participants’ LTPA before being diagnosed with GDM was based on twelve questions provided in the PPAQ (Supplementary table 1). The women were asked to assess how much time they spent in the course of a week on the respective activities, and this was subsequently divided into six categories: none; less than thirty minutes; more than thirty minutes, but less than one hour; between one and two hours; between two and three hours; and more than three hours. The categories were then translated into durations: 0, 0.25, 0.75, 1.5, 2.5 and 3, and computed into average weekly time spent on each activity. The intensity was calculated based on the specific MET values assigned to each activity as shown in Supplementary table 1 [28]. Based on the total number of MET minutes per week, the women were classified as either fulfilling the recommended minimum level of physical activity of ≥600 MET minutes per week or not fulfilling the recommendations with <600 MET minutes. The PPAQ also measured non-leisure activities, such as cleaning and grocery shopping. The participants were asked to assess the number of hours per week spent on performing different housework tasks, heavy workloads, activities other than LTPA (for example walking/cycling to places) and sedentary time during pregnancy (average hours per day watching TV/using a computer outside of work/working on the computer, reading books, driving) [27].

Background variables were recoded as shown in tables 1 and 2. The gestational age was reported in weeks. The perceived health score was reported on a scale from 0 to 100, with 100 being the best health imaginable. Symptoms of depression were assessed using the Edinburgh Depression Scale-5, using a cut-off of ≥7 to indicate depressive symptoms [29]. Perceived pain was based on questions: I have no pain or discomfort, I have slight pain or discomfort, I have moderate pain or discomfort, I have severe pain or discomfort and I have extreme pain or discomfort. Women who answered moderate or severe pain or comfort were in the perceived pain group No women had extreme pain or discomfort. The women’s countries of birth were divided into six different categories: Norway; Western Europe and USA; Eastern Europe; Asia; Africa; and South-America, due to a broad variety of countries with a limited number of women from each.

**Table 1:** Socio-demographic characteristics in relation to sufficient and insufficient leisure time physical activity (LTPA), *N*=238

	**Sufficient LTPA** *N *= 106 (44.5)	**Insufficient LTPA** *N *= 132 (55.5)	***p*-value**
**Age**			0.100
< = 29	20 (18.9)	37 (28.0)	
30-37	65 (61.3)	63 (47.7)	
> = 38	21 (19.8)	32 (24.2)	
**Country of birth** n (%)			0.295
Norway	52 (49.1)	59 (44.7)	
Western Europe + USA	8 (7.5)	6 (4.5)	
Eastern Europe	12 (11.3)	9 (6.8)	
Asia	19 (17.9)	38 (28.8)	
Africa	12 (11.3)	18 (13.6)	
South America	3 (2.8)	2 (1.5)	
**Marital status** n (%)			0.649
Married/co-habiting	98 (92.5)	124 (93.9)	
Single/Other	8 (7.15)	8 (6.1)	
**Education** n (%)			0.202
Primary/lower secondary school/ No education	8 (7.5)	15 (11.4)	
Upper secondary school	22 (20.8)	35 (26.5)	
College or University < 4 years	24 (22.6)	35 (26.5)	
College or University ≥ 4 years	52 (49.1)	45 (35.6)	
**Main activity** n (%)			0.244
Employed or self-employed	84 (79.2)	99 (72.7)	
Not employed nor self-employed	22 (20.8)	36 (27.3)	
**Joint income** n (%)			0.021
≤ NOK 599,000	29 (27.4)	50 (37.9)	
NOK 600,000-799,000	10 (9.4)	20 (15.2)	
NOK 800,000-999,000	24 (22.6)	26 (19.7)	
≥ NOK 1,000,000	26 (24.5)	13 (9.8)	
Don’t know	17 (16.0)	23 (17.4)	
**Economic hardship** *n *= 232 (%)			0.090
No	43 (41.7)	40 (31.0)	
Yes	60 (58.3)	89 (69.0)	
**Language** n (%)			0.039
Native speaking Norwegians	56 (52.8)	52 (39.4)	
Non-Native speaking	50 (47.2)	80 (60.6)	
**Years lived in Norway**			0.302
< = 10 years	38 (35.8)	39 (29.5)	
More than 10 years	68 (64.2)	93 (70.5)

**Table 2:** Health and pregnancy related characteristics in relation to sufficient and insufficient leisure time physical activity (LTPA), *N*=238

	**Sufficient LTPA** *N *= 106 (44.5)	**Insufficient LTPA** *N *= 132 (55.5)	***p* value**
**Gestational age** mean (SD)	26.9 (5.3)	26.6 (4.7)	0.509
**Motivated**			0.001
No	7 (6.6)	29 (22.0)	
Yes	99 (93.4)	103 (78.9)	
**Parity** n (%)			0.116
Primiparous	55 (51.9)	55 (41.7)	
Multiparous	51 (48.1)	77 (58.3)	
**Perceived health score (0-100)** Mean (SD)	74.9 (18.4)	68.1 (20.0)	0.007
**Pre-pregnancy body Mass Index** *n *= 234 (%)			0.928
<24.9	47 (45.2)	54 (41.5)	
25-29.9	32 (30.8)	42 (32.3)	
30-34.9	15 (14.4)	22 (16.9)	
35-45	10 (9.6)	12 (9.2)	
**Tobacco** n (%)			0.230
No	105 (99.1)	123 (96.9)	
Yes	1 (0.9)	5 (3.8)	
**EDS-5**^**a**^ **(*n *= 226)**			0.117
Score < 7	86 (86.9)	101 (79.5)	
Score ≥ 7	13 (13.1)	26 (20.5)	
**Perceived pain**			0.027
No	37 (34.9)	29 (22.0)	
Yes	69 (65.1)	103 (78.0)	
**Walking problems**			0.189
No	66 (62.3)	71 (53.8)	
Yes	40 (37.7)	61 (46.2)	

^a^***EDS***: Edinburgh Postnatal Depression Scale Short version with 5 questions

## Analysis

For categorical variables, cross-tabulations with Pearson’s chi-square tests were used to calculate percentages and assess differences in socio-demographic characteristics, and health and pregnancy related characteristics, in relation to sufficient and insufficient LTPA. A student’s t-test was used to compare continuous variables. Bivariate logistic regression analysis was used to examine the predictors for not following the recommendation of ≥600 MET minutes per week (sufficient/insufficient LTPA variable). Univariable models were performed first, with the LTPA variable as the dependent variable. All variables in the preliminary univariable models were included in a multivariable model if associated with LTPA at a significant level of <0.1 in crude analysis. The full model contained the independent variables age, joint income, language, perceived health, perceived pain, economic hardship, parity and motivation. The variables were tested for multi-collinearity and high inter-correlation between variables was not found.

All statistical analysis were performed with SPSS for IBM statistical software package version 26 (IBM Corporation, Armonk, NY, USA). A two-sided P-value of ⩽0.5 was considered statistically significant.

## Results

A total of 238 women were included in the study. Table 1 shows the socio-demographic characteristics in relation to sufficient and insufficient leisure time physical activity (LTPA). Less than half of the women in the sample (44.5%) had sufficient LTPA according to the minimum of ≥600 Met minutes per week (Table 1). The sample included 108 native speaking women and 130 non-native Norwegian speakers. More native Norwegian speaking women and women with the highest joint family income had sufficient LTPA.

The majority of women in this study were motivated to be physically active during pregnancy (84.9%). Significantly more women in the sufficient LTPA group were motivated compared to those in the insufficient group, at 93.4% and 78.9%, respectively (Table 2). When asked why they did not exercise, the majority answered that it was due to pregnancy complications (52.8%). The other predominant reasons for not being active were ‘not motivated’ and ‘no time’ (22.2% and 22.6%, respectively) (data not shown in the tables). Missing data for this question was approximately 11%, indicating that some of the proposed reasons for inactivity had not been covered. There was no significant association between the reasons for not being active and sufficient/insufficient LTPA. Women in the sufficient LTPA group had a significantly higher perceived health score than women in the insufficient LTPA group (*p* = 0.007) (Table 2).

Table 3 shows different activities and mean Met minutes, based on LTPA, in relation to sufficient and insufficient LTPA. Women in the sufficient LTPA group reported spending more hours per week on activities other than LTPA and more hours of sedentary time per week. The mean MET minutes per week in the sufficient LTPA group was 1.704.2 (SD 1,094.7) compared to 279.6 (SD 183.9) in the insufficient LTPA group (Table 3).

**Table 3:** Different activities in relation to sufficient and insufficient leisure time physical activity (LTPA), *N*=238

	**Sufficient LTPA** *N *= 106 Mean (SD)	**Insufficient LTPA** *N *= 132 (SD) Mean (SD)	***p*-value**
**Heavy workload (h/week)**	12.5 (24.5)	7.6 (16.6)	0.084
**Other activity than reported exercise (h/week)**	7.5 (4.9)	5.4 (3.5)	<0.001
**Sedentary time (h/week)**	52.9 (20.07)	47.9 (23.3)	0.036
**Housework (h/week)**	37.3 (28.3)	33.8 (21.5)	0.309
**Mean Met minutes (based on LTPA)**	1.704.2 (1094.7)	279.6.4 (183.9)	>0.001

We then performed a logistic regression analysis to assess the impact of different factors on the likelihood of not having sufficient LTPA (Table 4).

**Table 4:** Logistic Regression Predicting likelihood for not having sufficient leisure time physical activity (LTPA) per week.

Characteristics	OR	*p*- value	AOR^a^	*p* -value
**Age**				
< = 29	1.90 (1.00-3.64)	0.049	1.87 (0.90-3.92)	0.99
30-37	ref		ref	
> = 38	1.57 (0.82-3.01)	0.173	2.18 (1.03-4.60)	0.041
**Joint income**				
≤ NOK 599,000	3.45 (1.53-7.73)	0.003	3.21 (1.17-8.85)	0.024
NOK 600,000-799,000	4.00 (1.45-10.98)	0.007	3.94 (1.26-12.304)	0.018
NOK 800,000-999,000	2.17 (0.91-5.15)	0.080	1.86 (0.73-4.72)	0.194
≥NOK 1,000,000	ref		ref	
Don’t know	2.71 (1.08-6.75)	0.033	1.86 (0.56-6.06)	0.305
**Language**				
Norwegian	ref		ref	
Non-Norwegian	1.72 (1.02-2.89)	0.039	1.65 (0.83-3.27)	0.154
**Perceived health score (0-100)**	0.98 (0.97-0.99)	0.008	0.98 (0.91-0.99)	0.029
**Economic hardship**	1.59 (0.93-2.74)	0.090	1.00 (0.51-1.89)	0.995
**Parity**				
Primiparous	ref		ref	
Multiparous	1.51 (0.90-2.53)	0.117	1.59 (0.87-2.90)	0.133
**Perceived pain**	1.91 (1.07-3.38)	0.028	1.37 (0.70-2.68)	0.353
**Motivated for activity**	0.251 (0.11-.60)	0.002	0.27 (0.11-0.68)	0.006

^a^controlled for the variables in the model (significant level <0.1 in crude analysis). Adjusted model: X^2^ (df 12, *n* =238 )= 39.67, *p* < 0.005, Cox & Snell R Square, 0.16) and Nagelkerke R Square 0.21, correctly classified 67.4%.

The women who were 38 years or older were twice as likely to have insufficient LTPA (AOR 2.18 95% CI 1.03-4.60). Those with a lower joint income were more than three times as likely not to follow the recommendations for sufficient LTPA (Table 4). Being motivated for physical activity and having a higher score on the perceived health scale decreased the odds of having insufficient LTPA. The full model was statistically significant, X^2^ (df 12, *n* =238) = 39.67, *p* < 0.005, indicating that it performed better than a model without the adjusting variables. The model as a whole explained between 15.7% (Cox & Snell R Square) and 21% (Nagelkerke R Square) of the variance, and the percentage accuracy in classification was 67.4%. .

## Discussion

Less than half of the women in this study had sufficient LTPA according to a recommended minimum of ≥600 MET minutes per week. In contrast, most of the women were motivated to be physically active during pregnancy (84.9%). Having a lower joint family income and being over 38 years of age increased the odds of them having insufficient LTPA. Women with sufficient LTPA scored higher on a perceived health scale.

The study found that 44.5% of the participants had sufficient LTPA, which is a higher proportion than found in the main body of literature on pregnant women’s level of physical activity [10–12, 30]. When interpreting the results of our study, it must be considered that women’s LTPA was self-reported and not objectively measured. Recall bias might also have influenced the reported levels of physical activity since we asked for their physical activity prior to being diagnosed with GDM, which might have been one or two weeks earlier. Our participants’ level of education was higher than that of the participants in other studies, which may have influenced their level of LTPA. The Finnish Gestational Diabetes Prevention Study (RADIEL) suggests that the risk of GDM can be reduced by approximately 40% by following a combination of moderate physical activity and diet intervention among high-risk women [31]. Although our study found that a higher proportion of women adhered to the recommendations than in the main body of the literature, more than half of the women nonetheless had insufficient LTPA. Thus, the promotion of physical activity is of utmost importance, especially among women at risk of GDM. Physical activity has long been prescribed to patients with diabetes due to improvements in blood glycaemia levels and insulin sensitivity [32]. Nearly one third of the 1,584 pregnant women participating in a study in the USA analysing the determinants of physical activity and provider advice during pregnancy did not receive advice about physical activity during prenatal care [12]. Provider advice was found to significantly increase women's intentions to meet physical activity recommendations in a mixed-method study conducted among 188 women in the USA in the second and third trimesters [33]. Midwives and health professionals involved in antenatal care play a prominent supportive role in promoting physical activity. However, midwives may also have a lower degree of understanding of physical activity recommendations [34]. According to a qualitative study conducted in Sweden, counselling on physical activity can be challenging for midwives as they strive to adjust the advice to the individual circumstances of each woman [35].

We did not find any significant differences in the participants’ level of LTPA with regards to women’s ethnicity and/or mother tongue when we controlled for age, income, perceived health/pain, parity and motivation. This is in contrast to other studies, which demonstrate lower levels of physical activity among immigrant groups [15, 16]. In another multi-ethnic Norwegian sample, 25% complied with the guidelines for physical activity, while this proportion was lower among South Asians (14%) and Middle Easterners (16%) compared with Westerners (35%) [10]. When interpreting our results, it must be considered that we have not validated the PPAQ in the Norwegian, Somali or Urdu language. However, the Norwegian women and native speaking Somali and Urdu women checked the suitability and assurance of the translated questionnaires prior to the study [24, 36].

An important finding from our study was that the women with sufficient LTPA had higher scores of perceived health. Most likely, this indicates physical health as we did not find significant differences in women’s EDS score, measuring anxiety and depression. Improved perceived health is in accordance with the findings of a systematic review on the effectiveness of physical activity interventions on pregnancy-related outcomes among pregnant women, which demonstrated a positive effect of such interventions on the well-being and physical and psychological health of pregnant women [37]. A study conducted among multi-ethnic pregnant women in Singapore showed that women with a lower level of perceived health were more likely to reduce their level of physical activity during pregnancy [16]. This message is important to communicate.

Interestingly, this study found that 93.4% of women with sufficient LTPA and 78.9% of women with insufficient LTPA were motivated to be physically active. It is important to consider that women were asked to report their current motivation for being physically active, and the recent GDM diagnosis may have been one reason for this motivation. A study conducted among 467 healthy pregnant women analysed women’s readiness to become or remain physically active according to the trans-theoretical model of change (TTM) [38]. Most of the participants classified as inactive showed a high motivational readiness or intention to increase their physical activity level. The results showed that receiving advice from health professionals to exercise during pregnancy increased their likelihood of being in stages 4–5 on the scale of readiness to change exercise habits, while higher age, multi-parity, pre-pregnancy overweight, unhealthy eating habits, pelvic girdle pain and urinary incontinence were more prevalent in women with low readiness to change their exercise habits (stages 1–3). Motivation for activity was a predictor of the women’s adherence to the national guidelines. Furthermore, significantly more women who had insufficient LTPA reported perceived pain (*p *= 0.027) which might be areason for why these women did not meet the recommendations.

## Conclusions and implications

A low joint family income and being over 38 years of age were factors associated with insufficient LTPA in pregnant women. Communication strategies to promote physical activity among pregnant women should be tailored towards women with low socio-economic backgrounds and promote the positive effects of physical activity on individually perceived health.

## Supplementary Information

**Additional file 1.** Questions regarding leisure time Physical activities in PPQA with answer options and MET values

## Data Availability

The datasets analysed in the present study are available from the corresponding author upon reasonable request.

## Abbreviations

GDM: Gestational diabetes mellitus
LTPA: Leisure-time physical activity
MET: Metabolic equivalent tasks
PPAQ: Pregnancy Physical Activity Questionnaire
RCT: Randomised controlled trial
