# Supplementary material for: Factors associated with the level of physical activity in a multi-ethnic pregnant population – a cross-sectional study at the time of diagnosis with gestational diabetes
Source: BMC Pregnancy Childbirth. 2022 Jan 3;22:1. doi: 10.1186/s12884-021-04335-x (PMC8722186; doi:10.1186/s12884-021-04335-x)
Supplement: Supplementary file 1 — Additional file 1. Questions regarding leisure time Physical activities in PPQA with answer options and MET values [file 12884_2021_4335_MOESM1_ESM.docx]

Supplementary table 1: Questions regarding leisure time Physical activities in PPQA with answer options and MET values.

| PPAQ  How much time a week have you spend for fun/exercise: | None | Less than 1/2  hour per week | ½ to almost 1  hour per week | 1 to almost 2  hour per week | 2 to almost 3  hours per week | 3 or more  hours week | **Met values (28) (28)** |
| --- | --- | --- | --- | --- | --- | --- | --- |
| Walking slowly | 0 | 0.25 | 0.75 | 1.5 | 2.5 | 3 | **3.2** |
| Walking fast | 0 | 0.25 | 0.75 | 1.5 | 2.5 | 3 | **4.3** |
| Walking up-Hill | 0 | 0.25 | 0.75 | 1.5 | 2.5 | 3 | **5.3** |
| Jogging | 0 | 0.25 | 0.75 | 1.5 | 2.5 | 3 | **7.0** |
| Prenatal exercise | 0 | 0.25 | 0.75 | 1.5 | 2.5 | 3 | **3.5** |
| Swimming | 0 | 0.25 | 0.75 | 1.5 | 2.5 | 3 | **4.9** ^b^ |
| Aerobic | 0 | 0.25 | 0.75 | 1.5 | 2.5 | 3 | **5.3** |
| Yoga/Pilates | 0 | 0.25 | 0.75 | 1.5 | 2.5 | 3 | **3.0** |
| Lifting weights | 0 | 0.25 | 0.75 | 1.5 | 2.5 | 3 | **3.5** |
| Cycling/Spinning | 0 | 0.25 | 0.75 | 1.5 | 2.5 | 3 | **6.8** |
| Other exercise activities* | 0 | 0.25 | 0.75 | 1.5 | 2.5 | 3 | **4.4*** |

*Mean of the other Met values, ^b^ mean of met values for swimming (6.0), water aerobics (5.3) and water gymnastics (3.5)
